# Tricin-enriched *Zizania latifolia* ameliorates non-alcoholic fatty liver disease through AMPK-dependent pathways

**DOI:** 10.1007/s10068-023-01311-3

**Published:** 2023-06-30

**Authors:** Bo Yoon Chang, Jin Hye Bae, Cho Young Lim, Yoon Hee Kim, Tae Young Kim, Sung Yeon Kim

**Affiliations:** 1 Institute of Pharmaceutical Research and Development, College of Pharmacy, Wonkwang University, Iksan, 54538 Jeonbuk Korea; 2 Technology Development Center, BTC Corporation, Ansan, 15588 Korea

**Keywords:** Tricin, *Zizania latifolia*, Enzyme-treated, NAFLD, Lipogenesis

## Abstract

This study aimed to identify and elucidate the mechanism underlying the protective effect of tricin-enriched *Zizania latifolia (Z. latifolia*) extract (ETZL) against free fatty acid (FFA)-induced lipid accumulation in vitro and non-alcoholic fatty liver disease (NAFLD) induced by a high-fat diet and fructose diet (HFD/F) in vivo. ETZL treatment significantly lowered body weight gain and decreased adipose tissue, lipid, aspartate aminotransferase (AST), and alanine aminotransferase (ALT) levels in HFD/F-fed mice. ETZL acted on phosphorylated acetyl-CoA carboxylase (ACC) and anti-peroxisome proliferator-activated receptor α (PPARα) by activating the adenosine monophosphate-activated protein kinase (AMPK) pathway and inhibiting sterol regulatory element-binding proteins-1 (SREBP)/fatty acid synthase (FAS) signaling to inhibit de novo adipogenesis and increase fatty acid oxidation. In addition, treatment with ETZL increased nuclear factor erythroid-2-related factor 2 (Nrf2) levels to activate the antioxidant pathway. FFA-induced oxidative stress and fatty acid accumulation in HepG2 cells confirmed the improvement in fat accumulation through the AMPK and Nrf2 pathway activities of ETZL. These results suggest that ETZL ameliorates NAFLD by regulating lipid metabolism and defending against oxidative stress via AMPK-dependent pathways.

## Introduction

Fatty liver is a disease in which 5% or more of the fat in the liver accumulates abnormally as white on abdominal ultrasonography. The stages of fatty liver disease can be classified according to the degree of fat mass accumulation (Singh et al., 2017). The incidence of non-alcoholic fatty liver disease (NAFLD) is increasing and is believed to be the cause of chronic liver disease. NAFLD is often accompanied by obesity, diabetes, and hyperlipidemia. Fatty liver can also occur due to various drugs, including female hormones or steroids, or suddenly losing weight (Shoreibah et al., 2016; Singh et al., 2017). Obesity is the most likely risk factor for NAFLD, and the likelihood of developing non-alcoholic steatohepatitis (NASH) increases proportionally with obesity severity. Fat accumulation leads to metabolic disorders, excessive mitochondrial reactive oxygen species production, and endoplasmic reticulum stress, leading to inflammation, cell damage, and cell death. It is necessary to manage health through the intake of functional foods that can prevent obesity and fatty liver, along with fundamental efforts such as weight control and regular exercise (Masarone et al., 2018). Presently, treatment focuses on controlling medical problems and disorders causing fatty liver, including dietary modification, exercise, and weight loss. New medicines, such as vitamin E and metformin, have been introduced to treat fatty liver disease (Li et al., 2013). In patients with NASH, confirmed by biopsy, a high dose of vitamin E (800 IU/day for 12 months) can be used as a therapeutic agent to improve liver tissue findings and steatohepatitis (Singh et al., 2017; Gluchowski et al., 2017). However, a study by Klein et al. involving more than 30,000 healthy men reported considerably increased prostate incidence and mortality in the vitamin E (400 IU/day, 7–12 years) group, raising concerns about safety (Klein et al., 2011). Due to these concerns, caution must be exercised when administering drugs over a lengthy period. It was once believed that metformin, which improves insulin resistance in the liver and muscles and inhibits the production of new fat in the liver by activating adenosine monophosphate-activated protein kinase (AMPK), would be beneficial for treating NASH. Research is currently being conducted using N-acetylcysteine (a glutathione precursor), betaine, and lipid thistle for their antioxidant effects by preventing lipid oxidation related to glutathione metabolism (Singh et al., 2017; Yang et al., 2019). However, no significant results have been reported in this regard. Therefore, safe, efficient, and economic anti-NAFLD agents are urgently needed.

Recent studies have investigated the potential for improving the alcoholic fatty liver using new approaches such as gut microbiome treatment (Kolodziejczyk et al., 2019) and nanoformulations (Zobeiri et al., 2018). However, most of these pharmacological agents are still at various stages of development. Natural products are easily accessible and do not require artificial synthesis; thus, they are beneficial for the effective management of NAFLD.

Manchurian wild rice has traditionally been dried or consumed as a tea with leaves, stems, and roots and fermented for use in various diseases. The scientific name of the Manchurian wild rice is *Zizania latifolia* Turcz. (*Z. latifolia*) (Chang et al., 2021). *Z. latifolia* is known for various pharmaceutical effects (Han et al., 2013; Moon et al., 2018; Yu et al., 2020; Yan et al., 2018). According to Jiang et al., the flavonoid, saponin, and phytosterol contents of Manchurian wild rice are 52.3, 12.1, and 4.3 times higher than white rice, respectively (Jiang et al., 2016). Jiao et al. reported tricin and flavonolignan groups in *Z. latifolia* (Jiao et al., 2007). Among them, tricin (5,7,4′-trihydroxy-3′,5′-dimethoxyflavone) occurs as a glycoside in rice bran and other grasses such as wheat, barley, and maize. Lee et al. reported in vitro antiobesity effects of tricin were confirmed in an HFD-induced obese animal model (Lee and Imm, 2018). Tricin is beneficial for HSC-targeted therapeutic and chemopreventive applications in hepatic fibrosis (Seki et al., 2012). Our previous study confirmed that the enzyme treatment time increased the tricin content in manchurian wild rice and standardized the extraction method (Moon et al., 2018; Chang et al., 2021). Tricin-enriched *Z. latifolia* extract (ETZL) showed a high antioxidant effect and improved alcoholic fatty liver (Chang et al., 2021). Therefore, we hypothesized that ETZL could effectively treat NAFLD and evaluated its efficacy.

## Materials and methods

### Preparation of ETZL

**Fig. 1:**
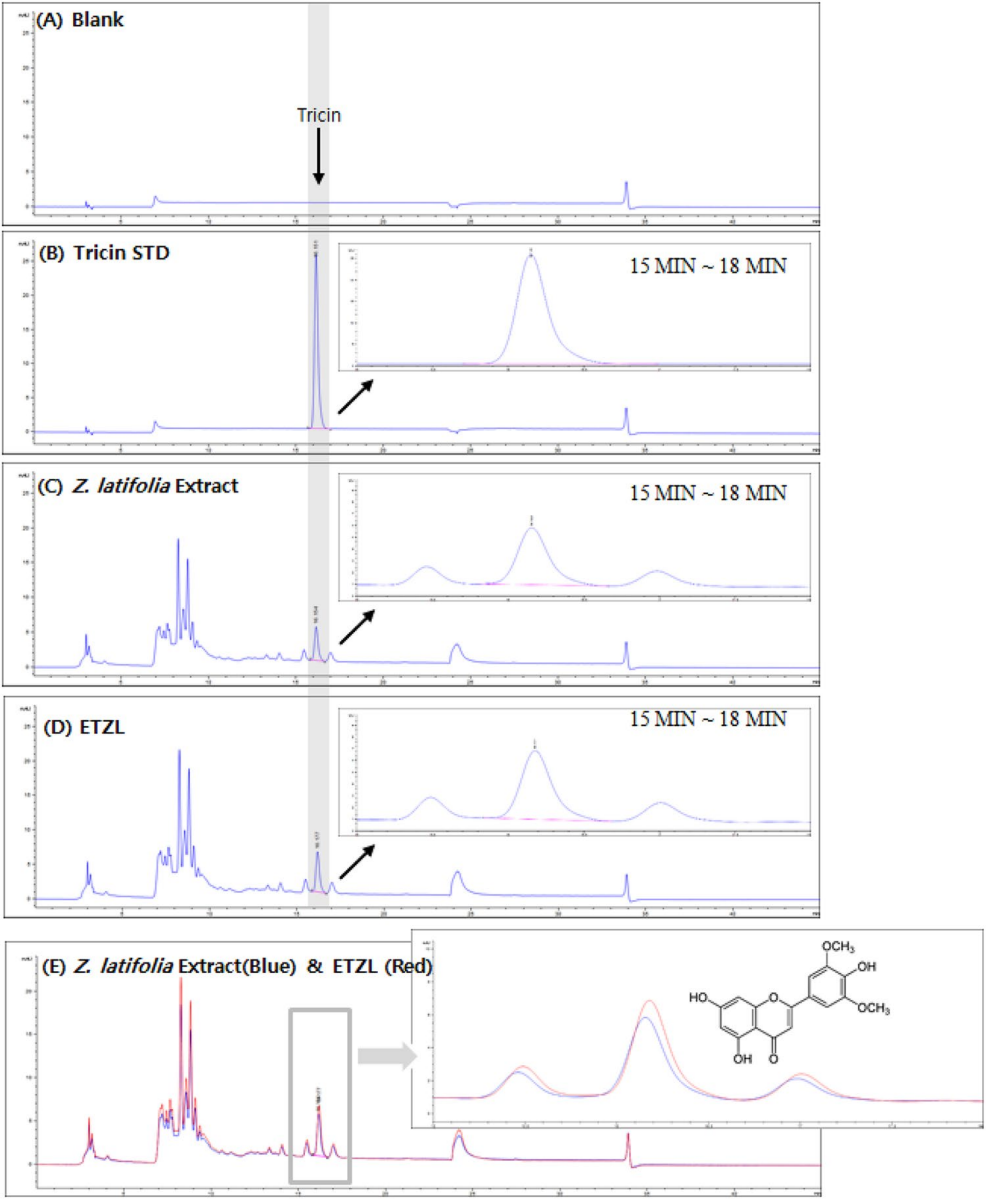
HPLC chromatograms of tricin contents. (**A**) HPLC chromatogram of blank; (**B**) HPLC chromatogram of tricin (standard); (**C**) HPLC chromatogram of *Z. latifolia* Extract; (**D**) HPLC chromatogram of ETZL; (**E**) Tricin peaks are merged *Z. latifolia* Extract (blue line) and ETZL(red line) on HPLC chromatogram

ETZL was provided by the BTC Corporation (Sangnok-gu, Ansan, Korea); it was extracted and treated with a mixed hydrolytic enzyme, as described previously (Moon et al., 2018). Experiments were conducted with ETZL from the same lot (BZI30-1910-01) used in our previously published study (Chang et al., 2021). Tricin was used as an ETZL marker for validation. Results of high-performance liquid chromatography (HPLC) of tricin content are shown in Fig. 1. The tricin content of *Z. latifolia* extract was 0.80 mg/g (Fig. 1C). After *Z. latifolia* extract enzyme treatment, the tricin content was 1.09 mg/g (Fig. 1D).

### Experimental animals and design

Five-week-old C57BL/6 male mice were obtained from Gbio Korea Inc. (Charles River, Gwangju, Korea). The animals were housed under optimal humidity (50–55%), and temperature (22–25 °C) with a 12 h light/dark cycle and were given free access to food and water. The Wonkwang University Animal Care Committee approved this study (WK20-45). The experimental diets used to induce NAFLD were a 60 kcal% fat diet and a 400 kcal% fructose; high-fat diet and fructose (HFD/F) diet (Rodent Diet D12091402; Research Diets, New Brunswick, NJ, USA). The mice in the Normal (NOR) group were fed a normal diet (ND; Rodent Diet D12450B; Research Diets). The mice were divided into NOR, HFD/F (HFD/F-CON), HFD/F + ETZL (100, 200, and 400 mg/kg), and HFD/F + metformin (200 mg/kg, positive control) groups. The ETZL concentration was chosen based on preliminary experiments and previous studies on the alcoholic fatty liver (Chang et al., 2021). Mice were divided into six groups (n = 8 per group). ETZL and metformin were administered orally. NOR or HFD/F received an identical volume of the vehicle.

During the test period, weight and food intake were measured weekly to calculate weight gain and food efficiency. Mice were anesthetized, and the amounts of fat and muscle in their bodies were measured and analyzed using pDEXA (InAlyzer DXA, Medikors, Seongnam, Korea) three times at four-week intervals. After the experiments, whole blood was collected, and the serum was separated. The wet weights of the harvested organs (liver and spleen) were measured. All samples were stored at − 80 °C until use.

### Biochemical assa*ys*

To evaluate liver function, aspartate aminotransferase (AST) and alanine aminotransferase (ALT) levels were measured by Frankel's method using serum samples. Serum triglyceride (Biovision, CA, USA), total cholesterol (Asan Co., Seoul, Korea), and high-density lipoprotein-cholesterol (Asan Co., Seoul, Korea) concentrations were quantified colorimetrically using enzymatic kits. Hepatic lipids were extracted using the Folch method, and triglyceride (TG) and cholesterol levels were measured in the same manner as in the serum. Serum leptin and adiponectin levels were measured using enzyme-linked immunosorbent assay kits (R&D Systems, Minneapolis, MN, USA). Malondialdehyde (MDA) was measured by the Ohkawa (1979) method using a thiobarbituric acid reaction (Ohkawa et al., 1979). Glutathione was measured using the Griffith (1979) method (Griffith et al., 1979).

### Histopathology

Hepatic tissues were fixed in a 10% buffered neutral formalin solution for 24 h and dehydrated with sucrose to prepare frozen blocks. Frozen sections were cut into 5 μm slices, pasted onto glass slides, and stained with Oil Red O and hematoxylin and eosin (H&E). A microscope at 200 × magnification was used to observe lipid accumulation in liver tissue. Quantitative staining was performed using the ImageJ software (NIH, USA).

### Gene expression analysis

Ribonucleic acid (RNA) was separated from each hepatic tissue sample using Easy Blue (iNtRON Biotechnology, Seongnam, Korea). The TaqMan one-step master mix (Applied Biosystems, Waltham, MA, USA) was used for reverse transcription and real-time polymerase chain reaction (PCR) of the messenger RNA (mRNA) samples. PCR was performed using the ABI 7500 real-time PCR system (Applied Biosystems, Foster City, CA, USA). FAS (Mm01204974_m1), sterol regulatory element-binding proteins-1c (SREBP1c) (Mm00550338_m1), heme oxygenase-1 (HO-1, Mm00516005_m1), NAD(P)H quinone oxidoreductase 1 (NQO1, Mm01253561_m1), tumor necrosis factor-alpha (TNF-α; Mm00443258_m1), interleukin-6 (IL-6; Mm00446190_m1), cyclooxygenase-2 (COX-2; Mm03294838_g1), and β-actin (Mm99999915_g1) were validated using the TaqMan Gene Expression Assay. Relative gene expression was calculated using StepOne software version 2.3 (Applied Biosystems, Foster City, CA, USA) with relative threshold cycle values. β-actin was used as the reference value. Three repetitions were performed for all animals for the harvested cell and hepatic samples of animals.

### Western blotting

PROPREP™ protein extraction solution (iNtRON Biotech, Seongnam, Korea) was used with dephosphorylation inhibitors to isolate proteins from cells or liver tissue. After separating 20 μg of protein by 10% SDS-PAGE, electroblotting with polyvinylidene difluoride membrane, blocking with 5% skim milk, primary antibody AMPK, anti-1 SREBP1c, anti-phospho-ACC (p-ACC), FAS, PPARα, anti-peroxisome proliferator-activated receptor gamma (PPARγ). After treatment with Nrf2, anti-HO-1, anti-NQO1, or anti-GAPDH or anti-β-actin antibodies (all antibodies were used at a 1:1000 dilution and purchased from Cell Signaling Technology, Danvers, MA, USA), the protein was detected using the enhanced chemiluminescence method. The protein blot intensities were analyzed using a FluorChem E imaging system (ProteinSimple; Santa Clara, CA, USA). In the case of the harvested cell sample, three repetitions were performed, and for the hepatic sample of animals, two repetitions were performed for all animals.

### Cell culture and lipid accumulation

HepG2 cells were purchased from the American Type Culture Collection (Manassas, VA, USA) and maintained in Dulbecco's Modified Eagle Medium 1% antibiotics and 10% fetal bovine serum in a humidified 5% carbon dioxide atmosphere at 37 °C. The cells used had fewer than five passages; HepG2 cells were incubated for 24 h in Dulbecco's Modified Eagle Medium containing 1 mM of a mixture of free fatty acids (FFA; oleic acid: palmitic acid = 2:1) and 1% FFA–free bovine serum albumin to induce intracellular lipid accumulation. For the ETZL experiments, HepG2 cells were treated with different concentrations of ETZL as indicated 2 h prior to the addition of the FFA mixture.

### Oil Red O staining of intracellular fat

FFA-treated cells were washed with phosphate-buffered saline and fixed with 4% paraformaldehyde for 1 h. After washing with 60% isopropanol, cells were incubated with Oil Red O for 20 min. The staining solution was washed with phosphate-buffered saline, and the cells were observed under a microscope. Oil Red O stain of the cells was dissolved in 100% isopropanol, and the lipid content was measured at 540 nm.

### Statistical analysis

Data are expressed as mean ± standard error. Significant differences were compared using repeated measures analysis of variance (ANOVA) followed by Duncan's multiple comparison test. Statistical significance was defined as *P* < 0.05. All statistical analyses were performed using GraphPad Software (GraphPad Software, Inc.).

## Results and discussion

### Effects of ETZL on anti-adipogenic activity in FFA-stimulated HepG2 cells

**Fig. 2:**
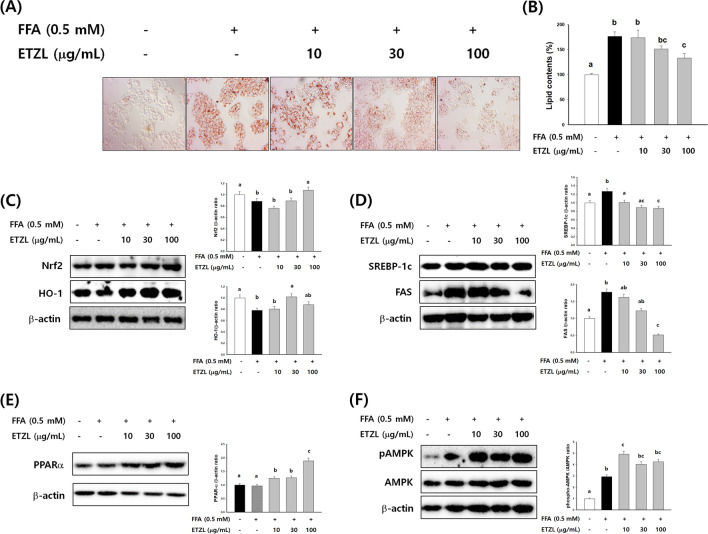
Effects of enzyme-treated *Zizania latifolia* (ETZL) on lipid accumulation in free fatty acid (FFA)-stimulated HepG2 cells. (**A**) The stained cells were observed under a microscope (200 ×). and (**B**) intracellular lipid levels were colorimetrically measured at 520 nm. (**C**) Nuclear factor erythroid-related factor (Nrf2), and Heme oxygenase 1 (HO-1), (**D**) sterol regulatory element-binding protein (SREBP)-1c, fatty acid synthase (FAS), (**E**) peroxisome proliferator-activated receptor alpha (PPARα) and (F) AMP-activated protein kinase (AMPK) protein levels were monitored by western blot analysis. Data are expressed as mean ± SE of three replicates. Values with different letters (a, b, c) are significantly different from one another (one-way ANOVA followed by Duncan's multiple comparison tests, *P* < 0.05)

Up to the experimental concentration of 100 μg/mL, cytotoxicity by ETZL treatment was not observed (data not shown). Treatment with 0.5 mM FFA using palmitic acid and oleic acid-induced oxidative stress and lipid accumulation in HepG2 cells, and these results were consistent with the study of Kim et al. (Kim et al., 2020). Palmitic acid is the most common saturated fatty acid in the blood, and various mechanisms of hepatotoxicity have been reported. Increased levels of fatty acids are metabolized in the mitochondria, peroxisomes, and endoplasmic reticulum, leading to oxidative stress (Fransen et al., 2017). FFA groups showed that the amount of Oil Red O staining increased 1.87 times compared to the NOR group without FFA treatment. The FFA + ETZL treatment group showed a significant dose-dependent decrease in the amount of Oil Red O staining compared to the FFA group (Fig. 2B, C). These results suggest that ETZL does not inhibit cell viability but reduces steatosis. *Z. latifolia* has strong antioxidant effects (Moon et al., 2018). To further investigate the relationship among ETZL, oxidative stress, and lipid accumulation, in the present research, ETZL was administered to FFA-induced HepG2 cells to observe the specific mechanisms of ETZL on lipid accumulation. ETZL was standardized by conducting a study to increase tricin content through the enzymatic treatment of *Z. latifolia* (Chang et al., 2021). Under oxidative stress, cells respond by activating the antioxidant defense machinery to combat cellular damage (Rejitha et al., 2015). We investigated the effect of ETZL on oxidative stress by analyzing changes in the Nrf2/HO-1 pathway. As shown in Fig. 2C, compared to the FFA group, incubation with ETZL increased the expression of Nrf2 and HO-1 in HepG2 cells (*P* < 0.01). These results suggested that ETZL reduced oxidative stress by increasing Nrf2 and HO-1 levels.

FFA treatment significantly upregulated FAS and SREBP-1c protein levels. However, ETZL markedly downregulated the expression levels of FAS and SREBP-1c. These results indicated that ETZL suppressed lipid accumulation by inhibiting the expression of lipogenic proteins such as SREBP-1c and FAS in HepG2 cells (Fig. 2D).

ETZL treatment significantly enhanced PPARα in FFA-exposed hepatocytes (Fig. 2E). Activation of PPAR-α induces uptake, utilization, and catabolism of fatty acids through the upregulation of gene expression involved in fatty acid transport, binding, and activation and increases mitochondrial fatty acid β-oxidation. ETZL treatment significantly increased the expression of phospho-AMPK (p-AMPK) in a concentration-dependent manner (Fig. 2F). AMPK, a master energy homeostasis regulator, is believed to play a central role in controlling lipid metabolism by modulating various metabolic proteins and enzymes (Zhang et al., 2021). AMPK also regulates Nrf2, a promising therapeutic target (Kim et al., 2020). These data indicate that AMPK activation mediates ETZL inhibition of lipogenesis and suppresses lipid accumulation in FFA-exposed hepatocytes.

### Effects of ETZL on body composition in NAFLD-induced mice

An HFD induces weight gain in mice and rats of various strains and results in diabetes, hyperlipidemia, and NAFLD. In the present study, NAFLD was induced in C57BL/6 mice, which were sensitive to HFD (Rossmeisl et al., 2003).

**Fig. 3:**
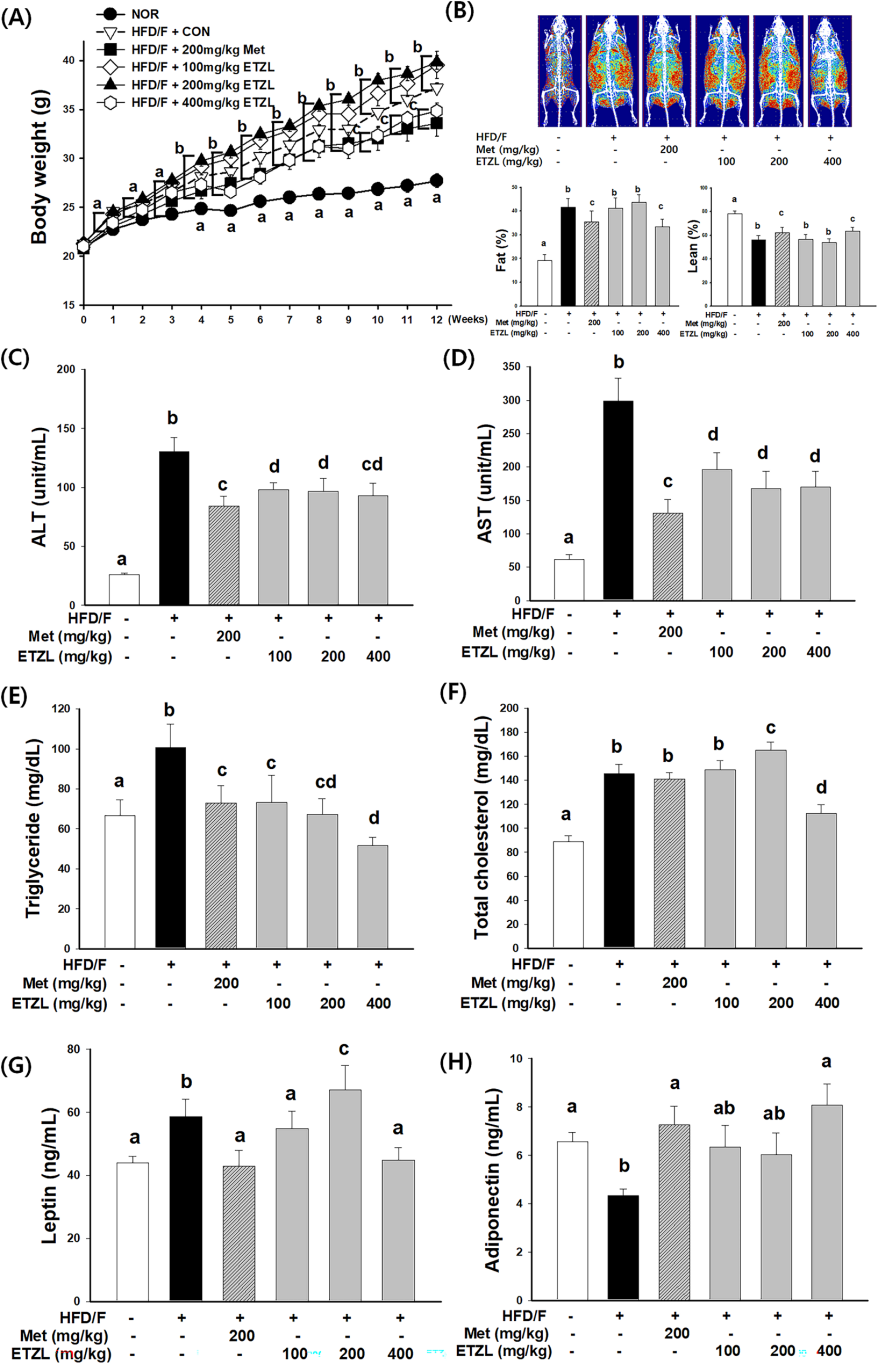
Effects of ETZL on body weight and composition and serum biochemistry in NAFLD-induced mice. (**A**) Weekly body weights over 12 weeks, (**B**) dual-energy X-ray absorptiometry images of fat distribution and fat/lean mass. (**C**) alanine transaminase (ALT), (**D**) aspartate transaminase (AST), (**E**) triglycerides, (**F**) total cholesterol, (**G**) leptin, and (H) adiponectin. Data are expressed as mean ± SE. n = 8, Values with different letters (a, b, c) are significantly different from one another (one-way ANOVA followed by Duncan's multiple comparison tests, *P* < 0.05)

The animals were fed an HFD/F freely to reduce stress. Mice fed an HFD/F did not show a significant difference in food intake (g/day/mouse) because they did not have a high preference for the taste or smell of HFD/F compared to standard mouse chow. However, the HFD/F (5.25 kcal/g) contained 40.9%p more calories than normal chow (3.10 kcal/g), and the total weight gain was significantly higher. Significant differences were observed after the fourth week of HFD/F diet-induced obesity. We found that the ETZL 400 mg/kg group showed a significantly decreased body weight compared to the HFD/F-CON group (Fig. 3A). Food and energy efficiencies also showed significant decreases in the ETZL 400 mg/kg group compared to the HFD/F-CON group (Table 1). During the 12th week, body composition analysis showed that fat increased by 2.1%, and lean mass decreased by 1.3% in the HFD/F-CON group. HFD/F-CON also has perirenal and epididymis fat of 6.8-fold and 4.7-fold, respectively, than NOR.

**Table 1:** Effects of ETZL on relative organ weight and food consumption in NAFLD-induced mice

Groups			Liver weight (%)	Spleen weight (%)	Gastrocnemius weight (%)	Perirenal fat weight (%)	Epididymal fat weight (%)	FER (%)	EER (%)
NOR			4.51 ± 0.15^a^	0.29 ± 0.03^a^	0.58 ± 0.03^a^	0.53 ± 0.09^a^	1.77 ± 0.21^a^	6.67 ± 1.44^a^	2.16 ± 0.46^a^
HFD/F	CON		3.33 ± 0.08^b^	0.19 ± 0.01^b^	0.44 ± 0.04^b^	6.27 ± 0.29^b^	23.56 ± 2.80^b^	4.50 ± 0.53^b^	2.75 ± 0.12^b^
	Met 200 (mg/kg)		3.88 ± 0.24^b^	0.24 ± 0.01^c^	0.49 ± 0.05^c^	5.26 ± 0.71^c^	18.95 ± 3.81^c^	4.02 ± 0.81^b^	1.95 ± 0.25^cb^
	ETZL (mg/kg)	100	3.31 ± 0.15^b^	0.20 ± 0.01^b^	6.51 ± 0.41^b^	23.48 ± 5.28^b^	4.48 ± 1.01^b^	0.42 ± 0.03^b^	2.49 ± 0.28^b^
		200	3.16 ± 0.03^b^	0.17 ± 0.00^b^	6.34 ± 0.09^b^	25.40 ± 3.06^b^	4.85 ± 0.58^b^	0.40 ± 0.04^b^	3.06 ± 0.06^b^
		400	3.26 ± 0.18^b^	0.26 ± 0.01^c^	5.01 ± 0.43^c^	19.69 ± 2.89^c^	3.76 ± 0.55^c^	0.51 ± 0.06^c^	1.95 ± 0.20^c^

*HFD/F* high-fat diet/fructose, *ETZL* tricin-enriched *zizania latifolia* extract, *FER* Food efficiency ratio, *EER* Eenergy efficiency ratio, *CON* Control, *NOR* Normal, *Met* metformin

Data are expressed as mean ± SE. n = 8

Values with different letters (a, b, c) are significantly different from one another (one-way ANOVA followed by Duncan's multiple range test, *P* < 0.05)

In contrast, in the ETZL 400 mg/kg group, compared to the HFD/F-CON group, fat decreased by 1.2%, and lean mass increased by 1.2% in the 12th week (Fig. 3B). From week 0 to week 12, the ETZL 400 mg/kg treatment group showed a decrease in body fat compared to the HFD/F-CON group and an increase in lean mass, except for fat. Understanding the biological behavior of an organ in response to changes in body weight is essential for evaluating organ weight. Normalization of organ weight to body weight helps eliminate variations because it is often helpful in clarifying treatment-related organ weight changes (Sellers et al., 2007). In another study that induced NAFLD with an HFD, the absolute liver weight increased, but the relative liver weight decreased, or there was no difference (Lau et al., 2017). In the present study, the relative liver weight was significantly lower in the HFD/F-CON group than in the NOR group. ETZL or metformin treatment did not alter the relative liver weight compared to the HFD/F-CON group. The relative weights of the spleen and gastrocnemius were markedly decreased in the HFD/F-CON group. These parameters were significantly improved by treatment with 400 mg/kg ETZL. The relative perirenal and epididymal fat weights did not differ between the HFD/F-CON and 100 and 200 mg/kg ETZL groups. However, the metformin and 400 mg/kg ETZL groups had lower perirenal and epididymal fat weights than the HFD/F-CON group.

Changes in body weight and composition demonstrated the effectiveness of HFD/F diet-induced obesity in mice. However, 400 mg/kg ETZL resulted in lower obesity induction than HFD/F-CON.

### Effects of ETZL on serum biochemistry in NAFLD-induced mice

The measurement of liver function-related enzymes, such as ALT and AST, can detect damage to hepatocytes and the biliary tract. In NAFLD, the activities of ALT and AST are elevated (Mashek, 2020; Singh et al., 2017). ALT is a hepatocyte-specific enzyme whose increased serum levels directly reflect liver damage. Oxidative stress during lipid deposition can damage hepatocyte mitochondrial membranes and reduce membrane fluidity. AST is released from the mitochondria of liver cells into the blood cells, increasing serum AST levels. Increased serum ALT and AST levels indicate a change from functional impairment to organic lesions. ALT and AST levels were measured as biochemical indicators of hepatotoxicity (Fig. 3C, D). The ALT and AST levels in the HFD/F-CON group showed 5.0- and 4.9-fold increases, respectively, compared to those in the NOR group. ALT and AST levels in the metformin group were significantly reduced by 35.5% and 56.2%, respectively, compared to those in the HFD/F-CON group. In the ETZL group, the concentrations of ALT in the blood at 100 and 400 mg/kg were significantly decreased by 24.9% and 28.5%, respectively, compared to the HFD/F-CON group. The serum AST concentration showed a significant decrease of > 4.3% at all ETZL doses. Serum triglyceride levels in the HFD/F-CON group increased by 51.1% compared to those in the NOR group. The metformin group showed a significant decrease of 27.7% compared with the HFD/F-CON group. In the 200 and 400 mg/kg ETZL groups, serum TG levels were significantly decreased by 33.2% and 48.6%, respectively, compared to those in the HFD/F-CON group (Fig. 3E). The total serum cholesterol of the HFD/F-CON group was increased by 63.6% compared to that of the NOR group, and the metformin group showed a significant decrease of 3.1% compared to that of the HFD/F-CON group. In the 400 mg/kg, compared to the HFD/F-CON group, total cholesterol showed a significant decrease of 22.7% compared to the HFD/F-CON group (Fig. 3F).

Leptin levels significantly increased, and adiponectin levels significantly decreased in the HFD/F-CON group compared to those in the NOR (Fig. 3G, H). According to a cohort study by Marques et al., serum leptin discriminates against NAFLD, and adiponectin combined with specific lipids stratifies NASH (Marques et al., 2021). Leptin may exert an anti-steatotic action in early NAFLD by promoting fatty acid oxidation and decreasing lipogenesis and a pro-inflammatory and pro-fibrotic action at later disease stages by increasing hepatic reactive oxygen species generation, pro-inflammatory cytokine release, and enhancing fibrinogenesis. Adiponectin regulates several metabolic functions, including glucose control and fatty acid oxidation. A reduction in adipocyte differentiation and an increase in energy expenditure associated with the mitochondrial uncoupling result from increased blood adiponectin concentration (Bauche et al., 2007). The present study showed that ETZL reduced serum leptin and increased serum adiponectin levels in HFD/F-fed mice. However, the food intake of the ETZL group was not significantly different from that of the HFD/F-CON group. Therefore, improved lipid metabolism after ETZL administration is likely related to the change in the concentration of these adipokines.

### Effects of ETZL on hepatic lipid contents and antioxidant activity in NAFLD-induced mice

**Fig. 4:**
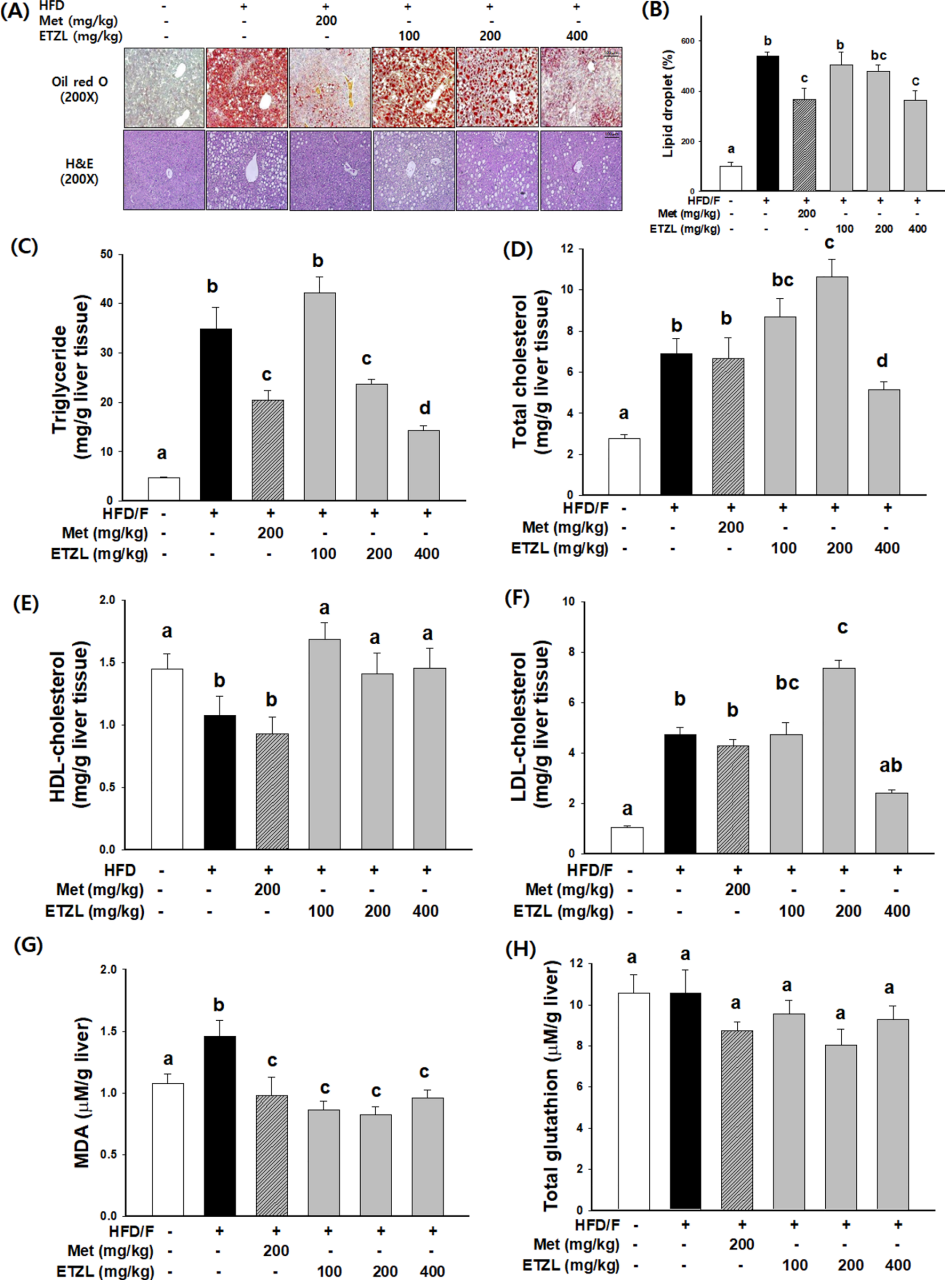
Effects of ETZL on hepatic lipid accumulation and antioxidant markers in NAFLD-induced mice. (**A**) Oil red O and H&E staining, (**B**) quantitative lipid droplets of oil red O staining hepatic sections, (**C**) hepatic triglyceride, (**D**) hepatic total cholesterol, (**E**) high-density lipoprotein-cholesterol (HDL), (**F**) low-density lipoprotein-cholesterol (LDL), (**G**) malondialdehyde (MDA), and (**H**) total glutathione. Data are expressed as mean ± SE. Independent experiments were performed in duplicate. n = 8, Values with different letters (a, b, c) are significantly different from one another (one-way ANOVA followed by Duncan's multiple comparison tests, *P* < 0.05)

**Fig. 5:**
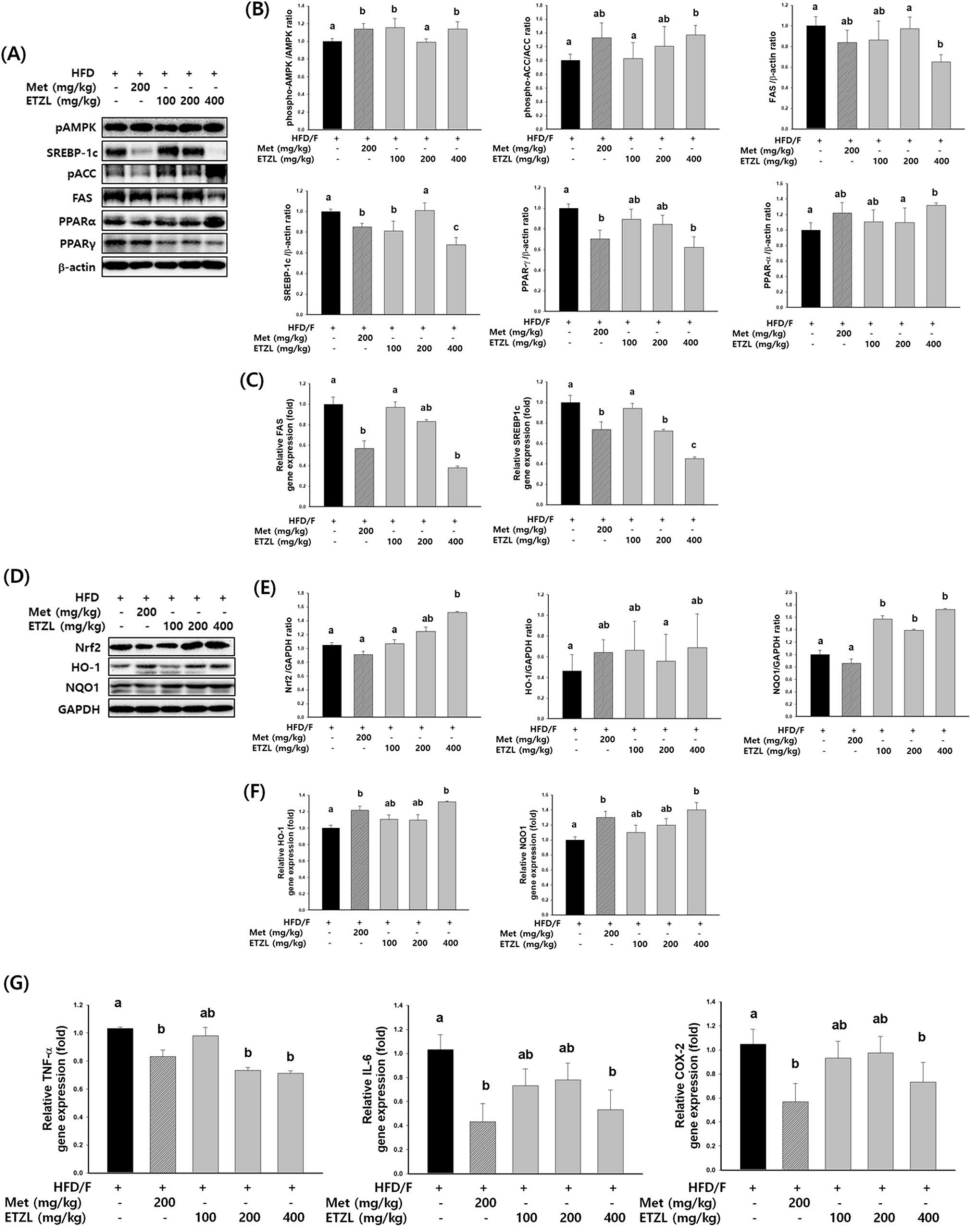
Effects of ETZL on lipogenesis, antioxidant, and anti-inflammatory in NAFLD-induced mice. (**A**) p-AMPK, SREBP-1c, p-ACC, FAS, PPARα, and PPARγ protein levels were monitored by western blot analysis, and (**B**) quantitative of protein densities. (**C**) The mRNA levels of FAS and SREBP-1c were monitored by real-time PCR. (**D**) Nrf2, HO-1, and NQO1 protein levels were monitored by western blot analysis and (**E**) quantitative protein densities. (**F**) The mRNA levels of HO-1 and NQO1 were monitored by real-time PCR. (**G**) The mRNA levels of TNF-a, IL-6, and COX were monitored by real-time PCR. Independent experiments were performed in triplication. n = 8, Data are expressed as mean ± SE. Values with different letters (a, b, c) are significantly different from one another (one-way ANOVA followed by Duncan's multiple comparison tests, *P* < 0.05)

Food fat is absorbed in the intestine, and blood lipids interact with or combine with synthetic lipoproteins to circulate in the blood. Therefore, serum and hepatic TG and TC levels are closely related to fatty liver. Representative Oil red O-and H&E-stained liver sections are shown in Fig. 4A. H&E, and Oil Red O staining demonstrated that induced NAFLD contributed to fatty liver degeneration, ballooning degeneration, and decreased infiltration of mixed inflammatory cells in the lobules of liver tissues, along with decreased neutral fat deposition. HFD/F treatment induces significant hepatic lipid accumulation. In the ETZL groups, the reduction in hepatic lipids increased by HFD/F was histologically and biochemically observed (Fig. 4). The content of hepatic TG, an essential lipid in NAFLD, increased 7.42-fold compared to that in NOR by HFD/F. The metformin group showed a significant decrease of 41.3% compared with the HFD/F-CON group. Hepatic TG levels in the 200 and 400 mg/kg ETZL groups decreased by 32.1% and 59.0%, respectively, in a dose-dependent manner (Fig. 4C). The hepatic total cholesterol level in HFD/F-CON mice was 2.46-fold higher than that in NOR mice. The total cholesterol content in the 400 mg/kg ETZL group did not change, but low-density lipoprotein cholesterol was reduced without altering the high-density lipoprotein cholesterol levels (Fig. 4D–F). These data suggested that ETZL modulated lipid accumulation in the liver. The hepatic MDA level of the HFD-CON group increased by 41.7% compared to that of the NOR group. The metformin group showed a significant decrease of 35.4% compared with the HFD/F-CON group. The hepatic MDA level of the HFD-CON group increased by 41.7% compared to that of the NOR group. The metformin group showed a significant decrease of 35.4% compared with the HFD/F-CON group. The hepatic MDA level in the ETZL group was significantly decreased by more than 36.6% compared to that in the HFD/F-CON group (Fig. 5G). No significant differences were observed in the hepatic glutathione by HFD intake, metformin treatment, or ETZL treatment (Fig. 4H).

### Effects of ETZL on anti-lipogenic activity in NAFLD-induced mice

We measured the levels of the proteins and mRNA involved in lipogenesis. SREBP1c, FAS, and PPARγ were decreased, and pAMPK, pACC, and PPARα were increased by ETZL or metformin administration (Fig. 5A, B). We measured the mRNA levels of the genes involved in lipogenesis. ETZL significantly reduced FAS and SREBP1c genes (Fig. 5C). SREBP-1c is a transcription factor that regulates the synthesis of fatty acids, cholesterol, and triglycerides; this promotes the expression of ACC and FAS, eventually leading to fatty acid synthesis. SREBP-1c and AMPK levels negatively correlate because AMPK activity modulates SREBP-1c inhibition. Several studies have shown that mice fed an HFD with FFA-induced HepG2 cells suppressed AMPK activity (Kim et al., 2020). In the present study, HFD/F-inactivated AMPK was upregulated by ETZL, which downregulated the expression of SREBP-1c, FAS, and ACC. PPAR-α, a major factor regulating β-oxidation, can potentially prevent NAFLD. NAFLD can inhibit the breakdown of free fatty acids by directly inhibiting the β-oxidation of FFA (Jorgačević et al., 2021); this leads to the accumulation of cytotoxic lipid substances (such as cholesterol) in the liver, resulting in liver cells and mitochondrial damage. Upregulation of PPAR-γ suggests that ETZL contributes to decreased β-oxidation, inhibiting lipid accumulation in hepatocytes.

AMPK acts as a switch in cellular metabolism, regulating changes between the anabolic and catabolic states (Zhang et al., 2021). In the liver, AMPK activates mitochondrial biosynthesis, fatty acid oxidation, and glucose production pathways and deactivates fatty acid synthesis, gluconeogenesis, and adenosine triphosphate consumption. AMPK plays a key role in metabolic liver disorders. PPAR-α and p-AMPK are well-known mediators in fatty acid metabolism that enhance metabolic processes. Disrupted fatty acid metabolism is involved in the pathogenesis of steatosis. Qu et al. reported that AMPK regulates Nrf2 translocation to induce HO-1 expression (Qu et al., 2016). Increasing evidence suggests that Nrf2 plays a vital role in fatty acid metabolism as a negative regulator of lipogenesis by activating antioxidant responses (Xu et al., 2018). ETZL significantly increased the expression levels of Nrf2, HO-1, and NQO1 proteins compared to those in the HFD/F-CON group (Fig. 5D, E). ETZL treatment significantly increased the mRNA expression of HO-1 and NQO1 (Fig. 5F). Cellular stress activates both AMPK and Nrf2. AMPK regulates Nrf2, which promotes the transcription of Nrf2 downstream antioxidant genes. Additionally, it has been demonstrated that activating the Nrf2 and AMPK pathways efficiently shields the liver from cellular oxidative stress. Therefore, the pharmacological activation of AMPK-Nrf2 expression via ETZL has the potential to attenuate the onset of fatty liver disease.

### Effects of ETZL on hepatic inflammation in NAFLD-induced mice

Since obesity is a chronic inflammatory condition that can damage the liver, liver inflammation was examined in each group. The expression of TNF-α and IL-6 were detected to assess the role of ETZL in the inhibition of inflammation. NAFLD is hypothesized to follow the two-hit theory, in which the first hit causes fat to accumulate in the liver, and the breakdown of adipose tissue enters the liver and releases FFA. After that, various oxidative stresses develop, and the second hit occurs owing to lipid peroxidation and inflammatory cytokine production, leading to steatohepatitis accompanied by hepatocyte damage and inflammatory response. (Singh et al., 2017). TNF-α plays a pivotal role in developing NAFLD and progression to NASH through upregulating key molecules associated with lipid metabolism, inflammatory cytokines, and fibrosis in the liver (Leyva-Jiménez and Ruiz-Malagón, 2020). The excessive increase of IL-1β and IL-6 aggravates inflammatory cytokines production and leads to hepatic steatosis and metabolic disorders. COX-2 is upregulated in patients with metabolic syndrome and animal models, accumulating prostaglandins (Lee et al., 2020). Herein, we found that ETZL suppressed the expression of pro-inflammatory cytokines in the livers of NAFLD mice, including TNF-α, IL-6, and COX-2 (Fig. 5G).

The HFD/F-induced NAFLD mouse model demonstrated that ETZL, whose tricin content was increased by enzymatic treatment, protected hepatocytes from NAFLD. The protective effect of ETZL can act by promoting the activation of AMPK signaling in lipids that partially increase fat degeneration. It has also been confirmed that ETZL administration suppresses oxidative stress and inflammation in the liver.

These results suggested that ETZL exerted protective effects against HFD/F-induced NAFLD in the liver by preventing lipid metabolism disorders. Thus, ETZL can be a potential therapeutic or preventive agent for fatty liver disease.

## Data Availability

The data used to support the findings of this study are available from the corresponding author upon request.

## Abbreviations

NAFLD: Non-alcoholic fatty liver disease
HFD/F: High-fat diet and fructose diet
FFA: Free fatty acid
ACC: Acetyl CoA carboxylase
PPARα: Peroxisome proliferator-activated receptor alpha
PPARγ: Peroxisome proliferator-activated receptor gamma
Nrf2: Nuclear factor erythroid-2-related factor 2
TNF‑α: Tumor necrosis factor-alpha
IL-6: Interleukin-6
COX-2: Cyclooxygenase-2
NASH: Non-alcoholic steatohepatitis
ROS: Reactive oxygen species
AMPK: Adenosine monophosphate-activated protein kinase
ALT: Alanine aminotransferase
AST: Aspartate aminotransferase
MDA: Malondialdehyde
Nrf2: Nuclear factor erythroid-related factor
HO-1: Heme oxygenase-1
FFA: Free fatty acid
FER: Food efficiency ratio
EER: Energy efficiency ratio
CON: Control
NOR: Normal
Met: Metformin

## References

[CR1] Bauche IB, El Mkadem SA, Pottier AM, Senou M, Many MC, Rezsohazy R, Penicaud L, Maeda N, Funahashi T, Brichard SM (2007). Overexpression of adiponectin targeted to adipose tissue in transgenic mice: impaired adipocyte differentiation. Endocrinology..

[CR2] Chang BY, Kim HJ, Kim TY, Kim SY (2021). Enzyme-treated *Zizania latifolia* extract protects against alcohol-induced liver injury by regulating the NRF2 pathway. Antioxidants..

[CR3] Fransen M, Lismont C, Walton P (2017). The peroxisome-mitochondria connection: How and why?. International Journal of Molecular Sciences..

[CR4] Gluchowski NL, Becuwe M, Walther TC, Farese RV (2017). Lipid droplets and liver disease: from basic biology to clinical implications. Nature Reviews Gastroenterology & Hepatology..

[CR5] Griffith OW, Anderson ME, Meister A (1979). Inhibition of glutathione biosynthesis by prothionine sulfoximine (S-n-propyl homocysteine sulfoximine), a selective inhibitor of gamma-glutamylcysteine synthetase. Journal of Biological Chemistry.

[CR6] Han S, Zhang H, Qin L, Zhai C (2013). Effects of dietary carbohydrate replaced with wild rice (*Zizania latifolia* (Griseb) Turcz) on insulin resistance in rats fed with a high-fat/cholesterol diet. Nutrients..

[CR7] Jiang MX, Zhai LJ, Yang H, Zhai SM, Zhai CK (2016). Analysis of active components and proteomics of Chinese wild rice (*Zizania latifolia* (Griseb) Turcz) and Indica Rice (Nagina22). Journal of Medicinal Food..

[CR8] Jiao J, Zhang Y, Liu C, Liu J, Wu X, Zhang Y (2007). Separation and purification of tricin from an antioxidant product derived from bamboo leaves. Journal of Agricultural and Food Chemistry.

[CR9] Jorgačević B, Vučević D, Samardžić J, Mladenović D, Vesković M, Vukićević D, Ješić R, Radosavljević T (2021). The effect of CB1 antagonism on hepatic oxidative/nitrosative stress and inflammation in nonalcoholic fatty liver disease. Current Medicinal Chemistry.

[CR10] Kim DE, Chang BY, Jeon BM (2020). SGL 121 attenuates nonalcoholic fatty liver disease through adjusting lipid metabolism through AMPK signaling pathway. International Journal of Molecular Sciences.

[CR11] Klein EA, Thompson IM, Tangen CM, Crowley JJ, Lucia MS, Goodman PJ, Minasian LM, Ford LG, Parnes HL, Gaziano JM, Karp DD, Lieber MM, Walther PJ, Klotz L, Parsons JK, Chin JL, Darke AK, Lippman SM, Goodman GE, Meyskens FL, Baker LH (2011). Vitamin E and the risk of prostate cancer: the selenium and vitamin E cancer prevention trial (SELECT). JAMA..

[CR12] Kolodziejczyk AA, Zheng D, Shibolet O, Elinav E (2019). The role of the microbiome in NAFLD and NASH. EMBO Molecular Medicine.

[CR13] Lau JKC, Zhang X, Yu J (2017). Animal models of non-alcoholic fatty liver disease: current perspectives and recent advances. The Journal of Pathology..

[CR14] Lee D, Imm JY (2018). Antiobesity effect of Tricin, a methylated cereal flavone. High-Fat-Diet-Induced Obese Mice..

[CR15] Lee MR, Kim JE, Park JW, Kang MJ, Choi HJ, Bae SJ, Choi YW, Kim KM, Hong JT, Hwang DY (2020). Fermented mulberry (*Morus alba*) leaves suppress high fat diet-induced hepatic steatosis through amelioration of the inflammatory response and autophagy pathway. BMC Complementary Medicine and Therapies.

[CR16] Leyva-Jiménez FJ, Ruiz-Malagón AJ (2020). Comparative study of the antioxidant and anti-inflammatory effects of leaf extracts from four different morus alba genotypes in high fat diet-induced obesity in mice. Antioxidants.

[CR17] Li Y, Liu L, Wang B, Wang J, Chen D (2013). Metformin in non-alcoholic fatty liver disease: a systematic review and meta-analysis. Biomedical Reports..

[CR18] Marques V, Afonso MB, Bierig N, Duarte-Ramos F, Santos-Laso Á, Jimenez-Agüero R, Eizaguirre E, Bujanda L, Pareja MJ, Luís R, Costa A, Machado MV, Alonso C, Arretxe E, Alustiza JM, Krawczyk M, Lammert F, Tiniakos DG, Flehmig B, Cortez-Pinto H, Banales JM, Castro RE, Normann A, Rodrigues CMP (2021). Adiponectin, Leptin, and IGF-1 are useful diagnostic and stratification biomarkers of NAFLD. Frontiers in Medicine (lausanne)..

[CR19] Masarone M, Rosato V, Dallio M, Gravina AG (2018). Role of oxidative stress in pathophysiology of nonalcoholic fatty liver disease. Oxidative Medicine and Cellular Longevity.

[CR20] Mashek DG (2020). Hepatic lipid droplets: a balancing act between energy storage and metabolic dysfunction in NAFLD. Molecular Metabolism..

[CR21] Moon JM, Park SH, Jhee KH, Yang SA (2018). Protection against UVB-induced wrinkle formation in SKH-1 hairless mice: efficacy of tricin isolated from enzyme-treated zizania latifolia extract. Molecules..

[CR22] Ohkawa H, Ohishi N, Yagi K (1979). Assay for lipid peroxides in animal tissues by thiobarbituric acid reaction. Analytical Biochemistry..

[CR23] Qu LL, Yu B, Li Z, Jiang WX, Jiang JD, Kong WJ (2016). Gastrodin ameliorates oxidative stress and proinflammatory response in nonalcoholic fatty liver disease through the AMPK/Nrf2 pathway. Phytotherapy Research.

[CR24] Rejitha S, Prathibha P, Indira M (2015). Nrf2-mediated antioxidant response by ethanolic extract of *Sida cordifolia* provides protection against alcohol-induced oxidative stress in liver by upregulation of glutathione metabolism. Redox Report.

[CR25] Rossmeisl M, Rim JS, Koza RA, Kozak LP (2003). Variation in type 2 diabetes–related traits in mouse strains susceptible to diet-induced obesity. Diabetes..

[CR26] Seki N, Toh U, Kawaguchi K, Ninomiya M, Koketsu M, Watanabe K, Aoki M, Fujii T, Nakamura A, Akagi Y, Kusukawa J, Kage M, Shirouzu K, Yamana H (2012). Tricin inhibits proliferation of human hepatic stellate cells in vitro by blocking tyrosine phosphorylation of PDGF receptor and its signaling pathways. Journal of Cellular Biochemistry..

[CR27] Sellers RS, Morton D, Michael B, Roome N, Johnson JK, Yano BL, Perry R, Schafer K (2007). Society of toxicologic pathology position paper: organ weight recommendations for toxicology studies. Toxicologic Pathology.

[CR28] Shoreibah M, Raff E, Bloomer J, Kakati D, Rasheed K, Kuo YF, Singal AK (2016). Alcoholic liver disease presents at advanced stage and progresses faster compared to non-alcoholic fatty liver diseas. Annals of Hepatology.

[CR29] Singh S, Osna NA, Kharbanda KK (2017). Treatment options for alcoholic and non-alcoholic fatty liver disease: a review. World Journal of Gastroenterology.

[CR30] Xu D, Xu M, Jeong S, Qian Y, Wu H, Xia Q, Kong X (2018). The role of Nrf2 in liver disease: novel molecular mechanisms and therapeutic approaches. Frontiers in Pharmacology.

[CR31] Yan N, Du Y, Liu X, Chu C, Shi J, Zhang H, Liu Y, Zhang Z (2018). Morphological characteristics, nutrients, and bioactive compounds of *Zizania latifolia*, and health benefits of its seeds. Molecules (basel, Switzerland)..

[CR32] Yang J, Fernández-Galilea M, Martínez-Fernández L, González-Muniesa P, Pérez-Chávez A, Martínez JA, Moreno-Aliaga MJ (2019). Oxidative stress and non-alcoholic fatty liver disease: effects of omega-3 fatty acid supplementation. Nutrients..

[CR33] Yu X, Chu M, Chu C, Du Y, Shi J, Liu X, Liu Y, Zhang H, Zhang Z, Yan N (2020). Wild rice (Zizania spp.): a review of its nutritional constituents, phytochemicals, antioxidant activities, and health-promoting effects. Food Chemistry.

[CR34] Zhang K, Shi Y, Huang C, Huang C, Xu P, Zhou C, Liu P, Hu R, Zhuang Y, Li G, Hu G, Guo X (2021). Activation of AMP-activated protein kinase signaling pathway ameliorates steatosis in laying hen hepatocytes. Poultry Science..

[CR35] Zobeiri M, Belwal T, Parvizi F, Naseri R, Farzaei MH, Nabavi SF, Sureda A, Nabavi SM (2018). Naringenin and its nano-formulations for fatty liver: cellular modes of action and clinical perspective. Current Pharmaceutical Biotechnology..

